# Personalized Adaptive Gabor Filtering with Three-Stage Semi-Supervised Domain-Adversarial Learning for Cross-Subject SSVEP Decoding

**DOI:** 10.3390/s26123694

**Published:** 2026-06-10

**Authors:** Junjun Guo, Xiaonan Pan, Ning Mi, Jianrui Zhang, Ting Huyan

**Affiliations:** 1 School of Mathematics and Information Engineering, Longdong University, Qingyang 745000, China; ningmi@mail.nwpu.edu.cn (N.M.); zhangjr@ldxy.edu.cn (J.Z.); 2 School of Intelligent Manufacturing, Longdong University, Qingyang 745000, China; panxiaonan007@163.com; 3 School of Life Science and Technology, Northwestern Polytechnical University, Xi’an 710072, China; huyanting@nwpu.edu.cn

**Keywords:** BCI, SSVEP decoding, G-AFB, TriS-DANN, semi-supervised domain adaptation, cross-subject transfer learning, lightweight calibration

## Abstract

Improving the decoding accuracy and information transfer rate (ITR) of steady-state visual evoked potential brain–computer interface (SSVEP-BCI) systems, while enhancing cross-subject generalization and reducing calibration cost, is essential for practical deployment. This study proposes an end-to-end framework that integrates adaptive filtering with semi-supervised domain adaptation. The framework incorporates a Gabor adaptive filter bank (G-AFB) to optimize time–frequency representations and extract features matched to individual neural responses. It also introduces a three-stage semi-supervised domain-adversarial neural network (TriS-DANN), which combines unsupervised pre-alignment and supervised fine-tuning to align cross-subject feature distributions and enable lightweight calibration. On the 1.0 s public benchmark dataset, G-AFB-tCNN achieved 89.13% accuracy, a 4.63 percentage-point improvement over its conventional filter-bank counterpart. On the 0.4 s in-house dataset, G-AFB-tCNN achieved 91.85% accuracy, a 3.22 percentage-point improvement over the conventional fixed filter bank. In transfer learning, TriS-DANN reached 86.60% accuracy using 0.4 s segments extracted from the stimulation period and only 23.07% of the available target-domain training/calibration trials, demonstrating higher efficiency and stability than conventional fine-tuning. These results support the proposed framework as a feasible route toward reliable, low-calibration SSVEP-BCI systems.

## 1. Introduction

Brain–computer interfaces (BCIs) directly connect the brain with external devices and provide new communication and control channels for patients with neurological disorders. They have shown considerable potential in assisted communication, neurorehabilitation, and intelligent device control, and have become an important research direction in neuroengineering [1,2,3]. BCI systems include several paradigms, such as motor imagery (MI), P300 potentials, and steady-state visual evoked potentials (SSVEPs). Although MI is intuitive, it usually requires long-term user training and often yields limited decoding accuracy; P300-based BCIs are relatively reliable but generally have a lower ITR. By contrast, SSVEP-based BCIs are well suited for high-performance systems because of their high ITR, high signal-to-noise ratio (SNR), and minimal training requirements [4,5]. In recent years, SSVEP-BCI applications have expanded from text spellers and intelligent wheelchairs [6,7] to emerging scenarios such as third-arm control and metaverse interaction [8,9].

The key to the SSVEP paradigm is the accurate decoding of frequency-specific information embedded in electroencephalography (EEG) signals [10]. Decoding methods have evolved from traditional signal-processing approaches to deep learning methods. Canonical correlation analysis (CCA) is widely used as a baseline because it requires no user-specific calibration and is computationally efficient. It identifies user intent by maximizing the correlation between EEG signals and pre-defined sine–cosine reference signals [11]. However, CCA mainly exploits fundamental-frequency information and does not adapt to individual differences in neural responses, resulting in limited decoding accuracy. Subsequent improvements have followed two main directions. The first exploits richer frequency-domain information. For example, filter-bank CCA (FBCCA) decomposes broadband signals into multiple harmonic subbands and significantly improves recognition accuracy without requiring calibration data [12]. The second direction optimizes spatial filters and templates to incorporate individual information. For example, extended CCA (eCCA) constructs individualized reference templates by averaging calibration trials [13], whereas task-related component analysis (TRCA) learns spatial filters that maximize inter-trial reproducibility [14]. The success of TRCA has inspired several variants, including ensemble TRCA and high-performance methods such as CORRCA and TSCORRCA [14,15]. Although these approaches are effective, they typically require a large amount of calibration data.

Despite their success, traditional methods share important limitations. Most are essentially linear models and therefore have limited ability to capture the complex nonlinear dynamics of EEG signals. Moreover, they often require relatively long time windows to extract stable features, which restricts real-time responsiveness [16]. In particular, the subband division in FBCCA, a widely used SSVEP-BCI method, still relies on manually designed fixed filters and therefore cannot provide personalized adaptation.

To overcome these limitations, researchers have increasingly adopted deep learning (DL) techniques. Deep neural networks can model nonlinear EEG dynamics and automatically learn discriminative features from short time windows in an end-to-end manner, thereby improving decoding performance [16]. Convolutional neural network (CNN)-based models are representative examples in this field [17]. For instance, tCNN demonstrated the feasibility of directly using raw time-domain signals for end-to-end SSVEP decoding [18]. EEGNet provides a compact and efficient CNN architecture based on depthwise separable convolution, reducing model complexity while maintaining competitive performance and showing potential for resource-limited deployment [10]. SSVEPFormer further combines CNN-based local feature extraction with Transformer-based self-attention to capture long-range dependencies and has achieved favorable performance on several benchmarks [19]. To improve decoding further, deep models have been combined with the filter-bank strategy. Methods such as FB-tCNN [18], FB-EEGNet [20], and FB-SSVEPFormer [19] decompose broadband signals into multiple subbands and feed subband-specific features into neural networks. Other studies have integrated CNNs with traditional algorithms, such as convolutional correlation analysis (Conv-CA), to combine deep feature representation with the interpretability of CCA [21].

Although these methods, particularly filter-bank-based deep models, have achieved promising results, their architectures reveal a potential limitation. In most current frameworks, including FB-tCNN, FB-EEGNet, and FB-SSVEPFormer, the front-end filter banks still use fixed-parameter filters from traditional signal processing, such as Butterworth or Chebyshev filters with preset cutoff frequencies. This combination of an advanced DL core and a fixed traditional front end is effective but may not be optimal. Given the substantial neural-response variability across subjects, a one-size-fits-all filtering strategy may limit the ability of deep models to learn individualized time–frequency features, thereby constraining further performance improvement. This raises the following question: can a learnable adaptive filter bank be designed so that its parameters are optimized jointly with the downstream deep network and customized for each user? To answer this question, we propose an end-to-end learnable Gabor adaptive filter bank (G-AFB) as the network front end. Gabor kernels were selected because they can provide favorable joint time–frequency resolution for non-stationary EEG signals [22]. By making the center frequency and bandwidth trainable, G-AFB can learn a user-specific spectral analysis strategy that matches individual neural response characteristics.

After feature extraction is optimized for individual users, another more challenging issue arises in practical deployment: cross-subject generalization. Because physiological and cognitive states vary across individuals, a model trained on one subject often performs poorly when directly applied to a new user, even with an adaptive front end. This weak generalization leads to a core barrier to practical BCI deployment: high individualized calibration cost. Adapting a model to each new user usually requires time-consuming data collection and model fine-tuning, which limits convenient deployment and large-scale use of BCI systems.

To address this issue, researchers have explored both traditional signal-processing and deep learning solutions. In traditional methods, source-subject data are used to assist decoding for a new target subject. Early work such as transferred-template CCA (tt-CCA) constructs a high-quality transfer template for a new user by averaging EEG data from source subjects, thereby improving zero-calibration performance [23]. Multi-subject learning methods, such as MS-ECCA and MS-eTRCA [24], extend this idea by learning cross-subject spatial filters rather than merely transferring templates, enabling acceptable performance with little or no calibration data. However, these methods remain essentially linear and have limited feature representation capability, leaving a performance gap relative to end-to-end deep learning approaches.

In deep learning, a common transfer strategy is fine-tuning, in which a general model is pre-trained on source-domain data and then partially or fully adjusted using limited calibration data from a new target subject [25]. However, fine-tuning depends strongly on the quantity and quality of target-domain calibration data. Unsupervised domain adaptation (UDA) provides an alternative by using unlabeled target-domain data to reduce domain discrepancies [26]. This idea has been applied in several EEG-related scenarios. For example, in cross-device adaptation between dry and wet electrodes, non-adversarial strategies such as subject-specific normalization and similarity-based validation selection have improved generalization [27]. In the more common cross-subject setting, adversarial approaches represented by domain-adversarial neural networks (DANNs) have been widely used. Recent frameworks, such as CSA-GSDANN, have applied DANN to ultra-short-window SSVEP decoding and achieved promising results [28].

Nevertheless, existing UDA methods still face challenges when applied to practical cross-device or cross-subject BCI systems. First, most methods follow a purely unsupervised paradigm and assume that aligning global data distributions is sufficient to eliminate domain differences. Such coarse distribution alignment may not capture subtle neural characteristics in SSVEP tasks, where individual variability is pronounced. Second, effective distribution alignment often requires a large amount of unlabeled target-domain data. Although unlabeled data are easier to obtain than labeled data, users still need to complete a long pre-recording session, which remains far from a plug-and-play BCI system.

To address these issues, we develop a decoding framework that integrates adaptive front-end feature extraction with a lightweight calibration transfer strategy.

Specifically, the proposed framework consists of the following two core components:

1. At the feature-extraction level, we design a learnable Gabor adaptive filter bank (G-AFB) as the network front end. Unlike traditional fixed-parameter filters, G-AFB uses trainable parameters and is jointly optimized with the downstream network in an end-to-end manner. Therefore, it can learn a personalized spectral analysis strategy that best matches each user’s neural responses and extracts more discriminative features.
2. At the transfer-learning level, we propose a three-stage semi-supervised domain-adversarial neural network (TriS-DANN), to address the high calibration cost in practical deployment. The framework first performs unsupervised domain-distribution pre-alignment and then uses a very small number of labeled target-domain samples for supervised fine-tuning. In this way, the model can be rapidly and efficiently adapted from existing subjects to a new user with minimal calibration data.

## 2. G-AFB and TriS-DANN Framework

### 2.1. Overall Framework

We propose an end-to-end framework for SSVEP decoding that integrates personalized adaptive feature extraction with efficient cross-subject transfer learning. The framework consists of two main components: a G-AFB, which serves as the front-end feature extractor, and a TriS-DANN, which enables lightweight calibration for cross-subject adaptation.

**Figure 1:**
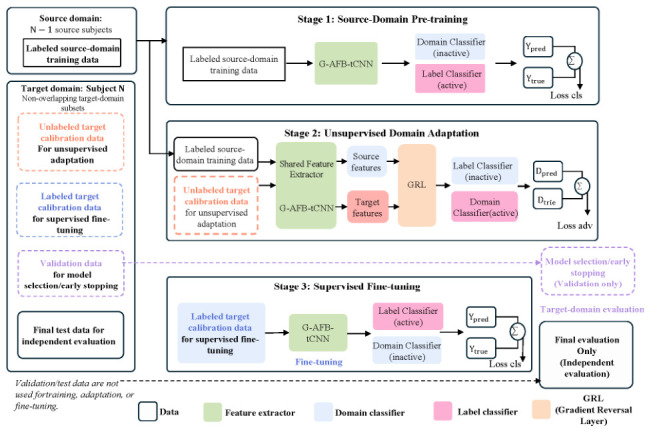
Overall workflow of the proposed framework. The target-domain data are divided into non-overlapping subsets for unsupervised adaptation, supervised fine-tuning, validation, and final testing.

Figure 1 illustrates the complete three-stage lightweight calibration procedure in a cross-subject setting, where the source domain consists of N−1 subjects and the target domain corresponds to one new subject.

Section 2.2 and Section 2.3 describe the two core components of the framework: the G-AFB model architecture and the TriS-DANN transfer strategy.

### 2.2. G-AFB: Learnable Gabor Adaptive Filter Bank Layer

#### 2.2.1. Gabor Kernels and Adaptive Mechanism

Unlike traditional approaches that use fixed filter banks, such as Butterworth or Chebyshev filters, we design a novel end-to-end learnable G-AFB layer based on trainable Gabor filters.

**Figure 2:**
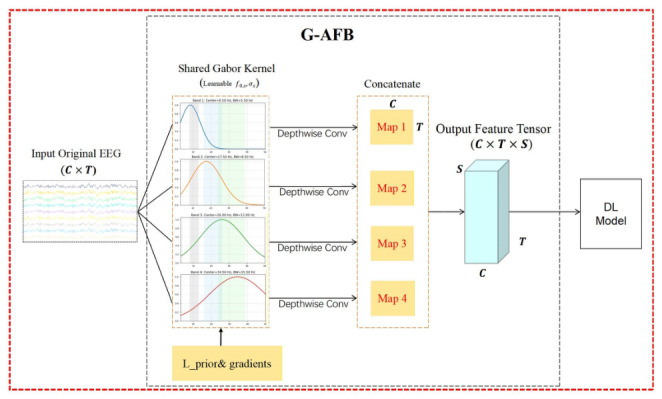
Workflow and architecture of the G-AFB layer. The learnable Gabor kernels extract harmonic-related subband features from multichannel EEG signals and feed the resulting features into the downstream decoding network. Different colors of the Gabor curves and feature maps indicate different harmonic-related subbands, and the light-blue cuboid denotes the concatenated output feature tensor.

The workflow and architecture of the G-AFB layer are shown in Figure 2. Given a raw multichannel EEG signal X∈R^C×T^, this layer performs parallel subband feature extraction using S shared learnable Gabor kernels. In this study, S = 4 corresponding to the first four harmonic-related subbands of the SSVEP stimulus frequencies. Here, “shared” means that the same set of Gabor-kernel parameters is applied across EEG channels and trials within the same model, rather than learning separate Gabor kernels for each channel or each trial. The Gabor-kernel parameters are optimized during model training and may differ across independently trained subjects, folds, or model instances.

For the s-th filter subband, the Gabor kernel g_s_(t) is defined in the time domain as a sinusoidal function modulated by a Gaussian envelope, where t denotes the local time index and s = 1, 2, …, S denotes the filter/subband index:

(1) $$g_{s}(t)=\exp\left(-\frac{t^{2}}{2\sigma_{s}^{2}}\right)\cos(2\pi f_{s}t),t\in \left[-\frac{K-1}{2},\frac{K-1}{2}\right]$$

This kernel function is determined by two learnable parameters: the center frequency f_s_ ∈ [3, 50] Hz, which specifies the spectral center of the subband of interest, and the subband bandwidth σ_s_ ∈ [0.5, 25] Hz, which determines the frequency selectivity of the subband. A smaller σ_s_ corresponds to a narrower frequency band. The kernel length K is dynamically determined as a proportion α of the input signal length T, thereby adapting to the analytical requirements of different time-window settings. In the implementation, fs and σs are treated as learnable parameters and updated by backpropagation together with the downstream network. To ensure physiological plausibility and numerical stability, fs and σs are constrained within the predefined ranges after each parameter update.

Subsequently, the input signal X is convolved with the S Gabor kernels g_s_ in the G-AFB layer using one-dimensional depthwise convolution, generating the feature map Y_s_ for each subband:

(2) $$Y_{s}=X{\;*}_{depthwise}\;g_{s},Y_{s}\in R^{C\times T}$$

Finally, the feature maps from all subbands are concatenated to form a frequency-enriched output feature tensor Y_G-AFB_, which is then fed into the subsequent network layer:

(3) $$Y_{\mathrm{G-}AFB}=[Y_{1},\ldots ,Y_{S}]\in R^{C\times T\times S}$$

#### 2.2.2. Loss Function Regularization

To constrain the learned Gabor-kernel parameters within physiologically meaningful ranges and accelerate convergence, we use a guidance mechanism that combines parameter initialization with prior-based regularization. Instead of random initialization, the initial parameters of each subband filter were determined according to the fundamental stimulus-frequency range of 8–13 Hz and its harmonic components.

Specifically, the EEG analysis range was 3–50~Hz, and the first four harmonics of the stimulus frequencies were considered; i.e., the harmonic order was set to n = 1, 2, 3, 4. In this study, the number of Gabor subbands was set to S = 4, corresponding to the first four harmonic ranges of the stimulus frequencies. For the n-th harmonic band, the initial center frequency was set to the theoretical center of the corresponding harmonic range, (fmin × n + fmax × n)/2, where fmin and fmax denote the minimum and maximum stimulus frequencies, respectively. Harmonic components outside the predefined EEG analysis range were excluded from the target-related frequency-band construction. Therefore, each learnable Gabor kernel was initialized around one target-related harmonic band, and its center frequency and bandwidth were further optimized during training through end-to-end backpropagation.

This initialization aligns the filters with the dominant spectral regions of SSVEP responses at the beginning of training. The prior regularization loss then serves as an anchor that prevents the learned filters from deviating excessively from these physiological priors during subject-specific fine-tuning, thereby improving convergence speed and training stability.

More importantly, the learning process of the G-AFB layer is driven by a composite loss function, as shown in Algorithm 1. This loss is composed of the classification loss L_cls_ and the prior regularization loss L_prior_:

(4) $$L_{total}=L_{cls}+L_{prior}$$

where L_prior_ is further composed of three components:

1. Frequency-alignment loss L_freq_: this term encourages the learned center frequencies {f_s_} to approach the key frequency bands {b_j_} associated with the SSVEP task:

(5) $$L_{freq}=\frac{1}{S}\sum_{s=1}^{S}\underset{j}{\min}|f_{s}-b_{j}{|}^{2}$$

2. Bandwidth-constraint loss L_bw_: this term penalizes bandwidth values σ_s_ that fall outside the predefined range of [2, 15] Hz. This range was selected based on physiological plausibility and empirical tuning to balance the coverage of individual SSVEP spectral variations and the suppression of unrelated EEG noise:

(6) $$L_{bw}=\frac{1}{S}\sum_{s=1}^{S}\left[{II}_{\sigma_{s}<2}(2-\sigma_{s})\cdot 10+{II}_{\sigma_{s}>15}(\sigma_{s}-15)\cdot 5\right]$$

3. Bandwidth-difference loss L_diff_: this term suppresses excessively large bandwidth differences among subbands and promotes smoothness in the filter bank.

(7) $$\left[L_{diff}=\frac{1}{S^{2}}\sum_{i=1}^{S}\sum_{j=1}^{S}\left|\sigma_{i}-\sigma_{j}\right|\right]$$

The final prior regularization loss is obtained as a weighted sum of the three components above:

(8) $$\left[L_{prior}=\lambda (L_{freq}+\alpha L_{bw}+\beta L_{diff})\right]$$

where $L_{freq,}\;L_{bw}$ and $L_{diff}$ control the relative contributions of frequency alignment, bandwidth constraint, and bandwidth-difference regularization, respectively. By using Ltotal as the optimization objective, the parameters of the G-AFB layer can be jointly optimized with those of the downstream decoding network in an end-to-end manner.
**Algorithm 1.** Forward Propagation and Loss Calculation of the G-AFB Layer.**Input:** $\;\mathrm{EEG}\;\mathrm{batch}\;X\in R^{N\times C\times T},$ $\mathrm{true}\;\mathrm{labels}\;Y_{true},$ downstream model DL_Model, filter number S, $\mathrm{target}\;\mathrm{bands}\;\{b_{j}\},$ weights λ,α,β **Learnable parameters:** $\{f_{s}\},$ $\{\sigma_{s}\},$ $\theta_{DL}$ **Output:**$\;\mathrm{Total}\;\mathrm{loss}\;L_{total}$1:$\mathrm{Initialize}\;\{f_{s}\},\{\sigma_{s}\}$ with heuristic rules2:For each filter s=1…S do 3:   $\;\mathrm{Generate}\;\mathrm{Gabor}\;\mathrm{kernel}\;g_{s}\left(t\right)$ $\mathrm{with}\;(f_{s},$$\sigma_{s}$)4:    Apply depthwise convolution of X $\mathrm{with}\;g_{s}\left(t\right),$ $\mathrm{obtain}\;Y_{s}$
5:End For6:$\mathrm{Concatenate}\;\{Y_{s}$$\}\;\mathrm{along}\;\mathrm{filter}\;\mathrm{dimension}\;\to \;Y_{\mathrm{G-AFB}}$7:$Y_{\mathrm{pred}}$ $\leftarrow \;\mathrm{DL}\_\mathrm{Model}(Y_{\mathrm{G-AFB}}$)8:$\mathrm{Compute}\;\mathrm{classification}\;\mathrm{loss}\;L_{\mathrm{cls}\;}$$\leftarrow \;\mathrm{CrossEntropy}(Y_{\mathrm{pred}},$ $Y_{\mathrm{true}}$)9:Compute prior losses: $L_{freq}=\frac{1}{S}\sum_{s=1}^{S}\underset{j}{\min}|f_{s}-b_{j}{|}^{2}$ $\left[L_{bw}=\sum_{s=1}^{S}(ReLU(2-\sigma_{s})+ReLU(\sigma_{s}-15))\right]$ $\left[L_{diff}=\frac{1}{S^{2}}\sum_{i=1}^{S}\sum_{j=1}^{S}\left|\sigma_{i}-\sigma_{j}\right|\right]$ $\left[L_{prior}=\lambda (L_{freq}+\alpha L_{bw}+\beta L_{diff})\right]$10:$L_{total}$ $\leftarrow \;L_{cls}$ $+\;L_{prior}$11:$\mathrm{Return}\;L_{total}$

During each forward pass, the Gabor kernels are generated from the current learnable parameters and then applied to the input EEG signals through depthwise convolution. Because the Gabor-kernel generation, convolution operation, and downstream classifier are included in the same computational graph, the gradients from both the classification loss and the prior regularization loss can be jointly backpropagated to the G-AFB parameters and the downstream network parameters. Therefore, the learned center frequencies and bandwidths are not fixed preprocessing parameters, but are optimized together with the decoding network during training.

#### 2.2.3. Integration with Downstream Networks

As a flexible front-end feature-extraction layer, G-AFB can be integrated with various mainstream deep learning decoding networks. To systematically validate its effectiveness, we constructed several models, including G-AFB-tCNN, G-AFB-EEGNet, and G-AFB-SSVEPFormer. The architecture of G-AFB-tCNN is shown in Table 1 as an example.

The G-AFB integration strategy follows the same principle for the other models. In G-AFB-EEGNet, the G-AFB layer is inserted before the initial two-dimensional convolutional filtering module of EEGNet. In G-AFB-SSVEPFormer, the G-AFB layer is placed before the patch-embedding module of the original SSVEPFormer. This flexible strategy enables the parameters of G-AFB to be jointly optimized with all downstream networks within a unified end-to-end framework.

### 2.3. Three-Stage Semi-Supervised Domain Adaptation Network (TriS-DANN)

After introducing the G-AFB-based architectures in Section 2.2, this section describes the core mechanism that enables lightweight calibration: the TriS-DANN. The main idea is to learn domain-invariant features through unsupervised distribution alignment and then perform efficient personalized fine-tuning using a small number of labeled target-domain samples.

The complete training procedure consists of three consecutive stages:

1. Source-domain pre-training

Using labeled source-domain data $D_{src}$ from multiple subjects, the feature extractor F and the label classifier C_y_ are jointly optimized to learn the basic time–frequency patterns of SSVEP signals and task-relevant generalizable features. The domain classifier C_d_ is not used in this stage.

2. Unsupervised domain alignment

After obtaining the general model, unlabeled target-domain data $D_{tar}^{u}$ are introduced and jointly used with the source-domain data for adversarial training. The domain classifier C_d_ learns to discriminate whether the features originate from the source or target domain, while the gradient reversal layer (GRL) reverses the domain-discrimination gradient and propagates it back to the feature extractor F, forcing F to generate domain-invariant feature representations. In this stage, the label classifier C_y_ is frozen.

3. Fine-tuning with a small number of labeled samples (lightweight calibration)

In the aligned feature space, a very small number of labeled target-domain samples $D_{tar}^{l}$ are used to jointly fine-tune F and C_y_, thereby enabling rapid and refined personalized calibration. To prevent overfitting, an independent target-domain validation set is retained during stages (b) and (c) for performance monitoring and early stopping, and the model with the best validation performance is finally saved.

The trainable modules differ across the three stages. In Stage 1, the feature extractor F and label classifier $C_{y}$ are optimized using labeled source-domain data, while the domain classifier $C_{d}$ is not used. In Stage 2, the label classifier $C_{y}$ is frozen, and adversarial training is performed using labeled source-domain samples and unlabeled target-domain samples. In this stage, the feature extractor F and domain classifier $C_{d}$ are updated through the gradient reversal mechanism, so that F learns domain-invariant representations while $C_{d}$ learns to distinguish source and target domains. In Stage 3, the domain classifier $C_{d}$ is no longer used, and the feature extractor F and label classifier Cy are fine-tuned using the small number of labeled target-domain calibration samples. The complete three-stage training procedure is summarized in Algorithm 2.
**Algorithm 2.** Training procedure of TriS-DANN.**Input:** Source domain data $D_{src}=\{(x_{i}^{src},\;y_{i}^{src})\}$ Target domain unlabeled data $D_{tar}^{u}=\{x_{j}^{tar}\}$ Target domain labeled data (few-shot)$D_{tar}^{l}=\{(x_{k}^{tar},\;y_{k}^{tar})\}$ Feature extractor $F(\cdot ;\theta_{F})$ Label classifier $C_{y}(\cdot ;\theta_{y})$ Domain classifier $C_{d}(\cdot ;\theta_{d})$ **Output:** Adapted feature extractor $\theta_{F},$ label classifier $\theta_{y},$ domain classifier $\theta_{d},$ Final adapted model M = {F, $C_{y}$}Step 1: Source-Domain Pre-training1:Initialize $\theta_{F},\theta_{y},\theta_{d}$
2:For minibatch $(x_{i}^{src},y_{i}^{src})∼D_{src}$ do3:    $f_{i}^{src}\leftarrow F(x_{i}^{src};\theta_{F})$
4:    $y_{pred}\leftarrow C_{y}(f_{i}^{src};\theta_{y})$
5:    Compute classification loss:                                           $\left[L_{label}=CrossEntropy(y_{pred},y_{i}^{src})\right]$
6:    Update $\theta_{F},\theta_{y}$ by minimizing $L_{label}$
7:End ForStep 2: Unsupervised Domain Adaptation8:For minibatch $(x_{i}^{src},\;y_{i}^{src})∼D_{src},$ minibatch $x_{j}^{tar}∼D_{tar}^{u}$ do9:    $f_{i}^{src}\leftarrow F(x_{i}^{src};\theta_{F}),$ $f_{j}^{tar}\leftarrow F(x_{j}^{tar};\theta_{F})$
10:Pass features through Gradient Reversal Layer (GRL)11:$d_{pred}^{src}\leftarrow C_{d}(f_{i}^{src};\theta_{d}),$ $d_{pred}^{\mathrm{tar}}\leftarrow C_{d}(f_{j}^{\mathrm{tar}};\theta_{d})$12:Compute domain loss:                                       $\left[L_{domain}=CrossEntropy(d_{pred}^{src},\;source\right)$                                               $+CrossEntropy(d_{pred}^{\mathrm{tar}},\;\mathrm{target})]$13:Update $\theta_{d}$ to minimize $L_{domain}$ (domain discrimination)14:Update $\theta_{F}$ via GRL to maximize $L_{domain}$ (domain confusion)15:End For(Note: $\theta_{y}$ frozen, only $\theta_{F},\theta_{d}$ updated)Step 3: Fine-tuning with Few Labeled Samples16:For minibatch $(x_{k}^{tar},y_{k}^{tar})∼D_{tar}^{l}$ do17:    $f_{k}^{tar}\leftarrow F(x_{k}^{tar};\theta_{F})$
18:    $y_{pred}^{tar}\leftarrow C_{y}(f_{k}^{tar};\theta_{y})$
19:    Compute fine-tuning loss:                                            $\left[L_{fine}=CrossEntropy(y_{pred}^{tar},y_{k}^{tar})\right]$
20:    Update $\theta_{F},\theta_{y}$ by minimizing $L_{fine}$
21:End For22:Return $\theta_{F},\theta_{y},\theta_{d}$


## 3. Experimental Setup and Results

To systematically evaluate the proposed framework, we first introduce the datasets used in the experiments (Section 3.1) and then conduct two main experiments. Experiment 1 uses within-subject evaluation to assess the ability of G-AFB to extract personalized features. Experiment 2 uses cross-subject evaluation to evaluate the generalization performance of TriS-DANN under a lightweight calibration setting.

### 3.1. Datasets

#### 3.1.1. Public Dataset

We used the widely adopted benchmark dataset acquired with a 40-target SSVEP-BCI speller [29]. The stimulus frequencies ranged from 8 to 15.8 Hz with an interval of 0.2 Hz, and joint frequency–phase modulation (JFPM) was used for coding. EEG data were collected using a 64-channel system and were downsampled to 250 Hz.

Data from 35 healthy participants were used. In the raw dataset, each participant completed six blocks, and each block contained the 40 visual targets presented in a random order; each trial lasted 5 s. Following the dataset recommendation, we selected eight occipital and parieto-occipital channels for analysis: O1, Oz, O2, PO3, Pz, PO5, PO4, and POz. Considering the 0.5 s visual cue and an approximate 0.14 s visual conduction delay, the EEG segment from 0.14 to 5.14 s after stimulus onset was extracted for analysis.

#### 3.1.2. In-House Dataset

To evaluate the model under conditions closer to real-world applications, we collected an in-house experimental dataset following the stimulus-design principles in [30]. The dataset included 10 healthy adult male participants aged 21–23 years (mean age: 22 years). All participants were right-handed, had normal or corrected-to-normal vision, and had no prior BCI experience. Before the experiment, all participants were fully informed of the procedure and signed informed consent forms.

Task Design and Stimulus Paradigm:

We designed a seven-class SSVEP experimental paradigm to evaluate decoding performance, particularly the ability to distinguish intentional commands from non-control states. The seven classes included six flickering stimuli at 8, 9, 10, 11, 12, and 13 Hz and one idle-state task representing the absence of intentional control. The idle-state stimulus was a static gray square, which was used to collect baseline EEG activity under non-task conditions and improve system robustness in practical applications.

**Figure 3:**
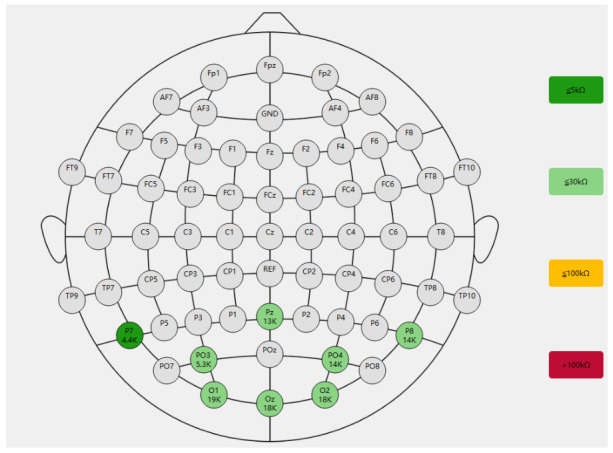
Impedance map of the selected eight-channel EEG acquisition montage used in the in-house experiment. Green circles indicate the selected EEG channels, with color intensity denoting the measured impedance range; gray circles indicate unselected electrode positions.

Visual stimuli were programmed using PsychoPy (version 2025.1.1) in Python 3.9 and presented on a 27-inch LCD monitor with a 165 Hz refresh rate to ensure stable stimulation frequencies. EEG signals were recorded using wet electrodes with conductive paste and an iRecorder W32 wireless EEG/ERP acquisition system from Shanghai Niantong Intelligent Technology Co., Ltd. Eight channels located in or near the occipital region were recorded: Oz, O1, O2, PO3, PO4, P7, Pz, and P8, as shown in Figure 3.

**Figure 4:**
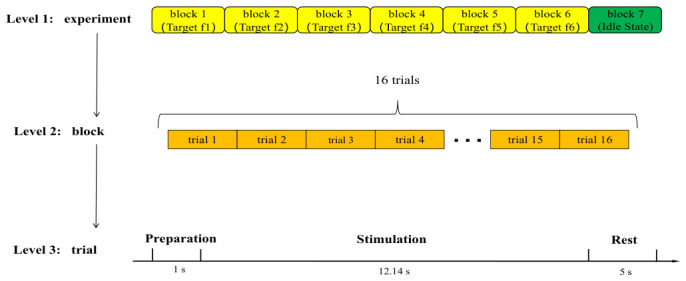
Experimental procedure of the in-house SSVEP task, including visual cue, stimulation period, and rest period. Yellow blocks indicate the six flickering target-frequency classes, the green block indicates the idle-state class, and orange boxes indicate repeated trials within each block. The ellipsis denotes omitted intermediate trials, and the arrows indicate the hierarchical and temporal progression of the experiment.

The experimental procedure is shown in Figure 4. The experiment consisted of seven task blocks, each corresponding to one of the seven classes, and the block order was pseudo-randomly balanced across participants. Each block contained 16 trials. In each trial, a 1 s visual cue first indicated the upcoming task, followed by a 12.14 s stimulation period and a 5 s rest period during which the screen turned black. Thus, each trial lasted 18.14 s. Considering an approximate 0.14 s visual conduction delay, the EEG segment from 0.14 to 12.14 s after stimulus onset was extracted as the analysis epoch, corresponding to a 12.0 s period of sustained visual stimulation.

### 3.2. Experiment 1: Validation of G-AFB Effectiveness (Within-Subject Evaluation)

#### 3.2.1. Experimental Setup

The purpose of this experiment was to validate the superiority of G-AFB over conventional fixed filtering under ideal conditions with sufficient subject-specific training data. All evaluations were conducted using a strict within-subject paradigm.

For the public benchmark dataset, we followed the conventional leave-one-block-out cross-validation strategy. For the in-house dataset, we adopted a more stringent leave-three-trials-out cross-validation strategy. All train/validation/test partitions were performed at the original trial/epoch level before applying the random-start sliding-window procedure. After data partitioning, short-window samples were generated independently within each subset. Consequently, all windows derived from the same original trial/epoch remained in the same subset, and no window from the test set was used for training, validation, model selection, or hyperparameter tuning.

**Figure 5:**
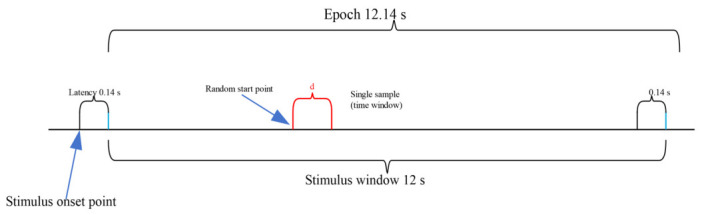
Random-start short-window construction. Trial/epoch-level partitioning was performed before window extraction, and random-start windows were generated independently within each subset. Blue arrows indicate the stimulus onset point and the random start point, the red bracket denotes the extracted single-sample time window of length d, and the cyan ticks mark the 0.14 s boundary positions.

Before training and testing, all data underwent a unified preprocessing and sample-construction procedure. Raw EEG signals were first downsampled to 250 Hz, band-pass filtered from 3 to 50 Hz, and re-referenced using common average reference (CAR). A random-start sliding-window strategy was used to construct short-window samples from each 12.0 s analysis epoch. For each predefined window length, the starting point was randomly selected within the analysis epoch, and the corresponding EEG segment was extracted as one sample (Figure 5). Importantly, these randomly selected short windows should not be interpreted as onset-locked early post-stimulus windows. Because the in-house experiment used a long-stimulation block design, a randomly selected 0.4 s or 1.0 s segment may come from a stabilized response period rather than from the first 0.4 s or 1.0 s after stimulus onset.

To clearly demonstrate the performance gain provided by G-AFB, we constructed a set of benchmark models. The core comparison kept the back-end deep decoding network unchanged while replacing only the front-end filtering module. Specifically, models equipped with G-AFB (G-AFB-tCNN, G-AFB-EEGNet, and G-AFB-SSVEPFormer) were compared with their fixed-filter-bank counterparts (FB-tCNN, FB-EEGNet, and FB-SSVEPFormer). In addition, classical decoding algorithms, including CCA and FBCCA, were included as traditional baselines.

All models were evaluated using two metrics widely adopted in BCI research. The first metric was classification accuracy, defined as the number of correctly predicted trials (C) divided by the total number of test trials (N). Because BCI systems are communication tools, transmission speed and efficiency must also be considered. Therefore, the information transfer rate (ITR), which jointly reflects decoding speed and accuracy, was calculated as follows:

(9) $$ITR=\left[\log_{2}Q\;+\;P\log_{2}P\;+\;\log_{2}\left(1-P\right)\log_{2}\frac{1-P}{Q-1}\right]\cdot \frac{60}{T}$$

where Q denotes the number of task classes, P denotes classification accuracy, and T denotes the analysis time-window length. In the public benchmark dataset, Q = 40; in the in-house dataset, Q = 7. To ensure fair and reproducible comparisons, all experiments were conducted on the same hardware platform, as listed in Table 2. The general hyperparameters for within-subject evaluation are listed in Table 3.

#### 3.2.2. Performance Evaluation on the Public Benchmark Dataset

We first performed a within-subject evaluation of G-AFB on the public benchmark dataset. The detailed classification accuracies are presented in Table 4, which compares the G-AFB-based models with several baseline methods across 35 subjects.

As shown in Table 4, G-AFB-tCNN achieved the highest mean accuracy (89.13%) among all compared models, providing preliminary evidence for the effectiveness of the proposed framework. To statistically examine the performance differences, we conducted one-way repeated-measures analysis of variance (ANOVA) followed by Bonferroni post hoc correction.

The statistical analysis showed that the effect of G-AFB varied across network architectures. For tCNN and SSVEPFormer, integrating G-AFB significantly improved performance and consistently outperformed both the corresponding fixed filter-bank versions and the no-filter baselines. These results demonstrate the effectiveness of G-AFB as a front-end processing module.

In particular, compared with the corresponding FB-tCNN model (84.50%), the best-performing G-AFB-tCNN model (89.13%) achieved a significant improvement of 4.63 percentage points (*p* < 0.01). This result indicates that G-AFB is advantageous for capturing individualized neural response features. Compared with manually designed fixed filter banks, this data-driven and end-to-end trainable adaptive filtering strategy can generate a more discriminative time–frequency feature extraction scheme for each user and thereby increase the performance ceiling of the decoding model.

An interesting exception was observed for EEGNet, for which the fixed filter-bank version (86.99%) outperformed the G-AFB version (82.53%). We speculate that this may be caused by incompatibility between the multichannel concatenated features generated by G-AFB and the compact internal convolutional structure of EEGNet, particularly its depthwise separable convolutions. This finding highlights the importance of co-designing the adaptive front end and the downstream decoding network, which is further discussed in Section 4.

#### 3.2.3. Performance Evaluation on the In-House Dataset with an Idle State

**Figure 6:**
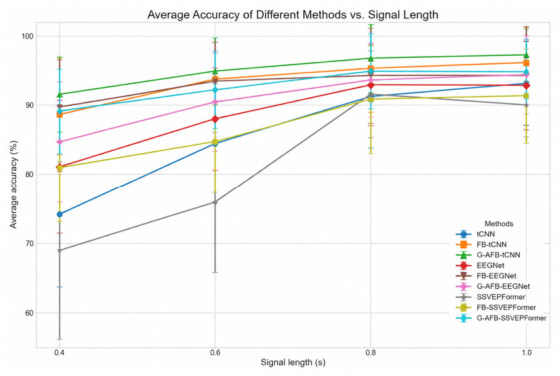
Average classification accuracy on the in-house dataset under different time-window lengths. Error bars indicate standard deviation.

**Figure 7:**
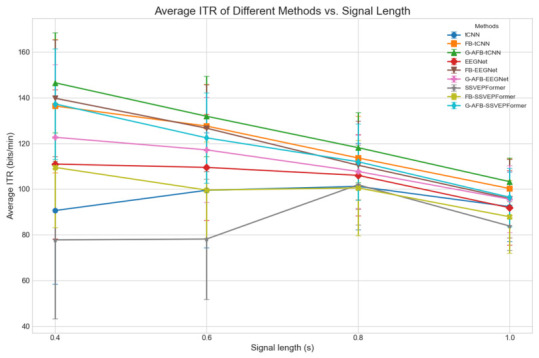
Average information transfer rate on the in-house dataset under different time-window lengths. Error bars indicate standard deviation.

To further evaluate the performance and robustness of G-AFB in a more practical scenario, we conducted experiments on the in-house seven-class dataset, which included an idle state. All models were tested using four time-window lengths: 0.4, 0.6, 0.8, and 1.0 s. Figure 6 and Figure 7 show the average classification accuracy and ITR of each model as a function of time-window length, respectively.

As shown in Figure 6, the accuracy of all models increased as the time window became longer, which is consistent with expectations. G-AFB-tCNN achieved the best performance under all tested conditions. In the most challenging 0.4 s segment setting, G-AFB-tCNN reached an average accuracy of 91.85%, clearly outperforming the other models and demonstrating strong classification performance for short segments extracted from stabilized SSVEP responses. Its ITR also peaked at 0.4 s (Figure 7), indicating a favorable balance between segment length and classification accuracy in the present offline analysis.

Further analysis of the G-AFB contribution showed results consistent with those obtained on the public dataset: G-AFB significantly improved both tCNN and SSVEPFormer. For example, at 0.4 s, G-AFB-tCNN (91.85%) outperformed FB-tCNN (88.63%) by 3.22 percentage points, and this advantage was maintained across all time windows. These results again confirm the superiority of personalized adaptive filtering over fixed filtering.

**Figure 8:**
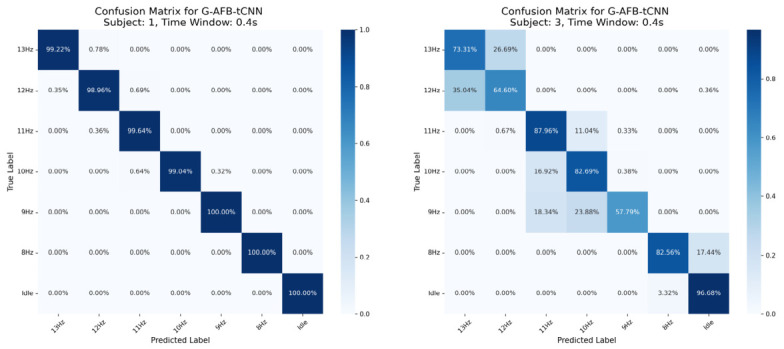
Confusion matrices of the best- and worst-performing subjects under the 0.4 s segment setting.

To further examine the ability of G-AFB-tCNN to distinguish different commands from the idle state, we selected two representative subjects: S1, who showed excellent overall performance, and S3, who represented a more challenging case. Their confusion matrices under the 0.4 s window are shown in Figure 8. For S1 (Figure 8, left), the model showed nearly perfect classification. The recall values of all six SSVEP command classes exceeded 98.9%, and the idle state was correctly identified in 100% of trials, with no false alarms. This indicates that the proposed framework can achieve highly accurate and reliable decoding when neural responses are of high quality. For S3 (Figure 8, right), although command decoding accuracy decreased and most errors occurred between spectrally adjacent frequencies, the ability to recognize the idle state remained robust, with an idle-state recall of 96.68%.

The marked performance difference between S1 and S3 highlights the substantial influence of individual variability on BCI systems. This difference is unlikely to be random; we infer that it is closely related to the intrinsic quality of the subjects’ raw EEG signals. To verify this inference, Section 3.2.4 introduces SNR as an objective metric for quantitative analysis. The key observation from the S3 confusion matrix is that, even under poor signal quality, the model retained robust idle-state recognition, which is critical for safe BCI deployment.

#### 3.2.4. Visual Analysis of the Individualized Filtering Mechanism of G-AFB

The experimental results above confirm the effectiveness of G-AFB. This section further examines its internal mechanism through visualization and addresses the following question: how does G-AFB achieve efficient personalized spectral feature extraction? We argue that its success lies in its ability to adapt to user-specific neural response patterns, which is necessary because of substantial physiological differences among users.

**Figure 9:**
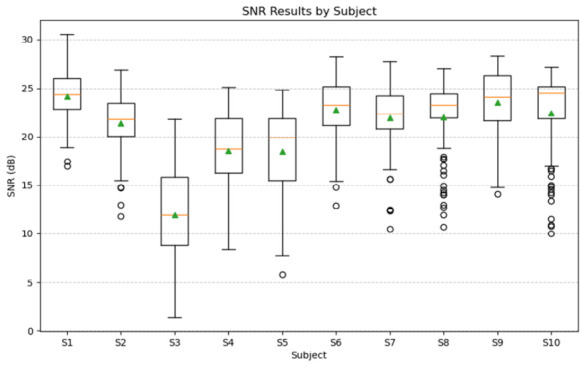
Box plot of average SNR across the ten subjects in the in-house dataset. In each box plot, the orange line indicates the median, the green triangle indicates the mean, the box represents the interquartile range, the whiskers indicate the non-outlier range, and open circles denote outliers.

First, we analyzed the SNR of SSVEP signals from different subjects in the in-house dataset to objectively demonstrate individual differences. As shown in the SNR box plot in Figure 9, signal quality varied substantially across subjects, providing physiological evidence for the need for personalized adaptation. A one-size-fits-all model designed for users with high SNR may perform poorly for users with low SNR.

**Figure 10:**
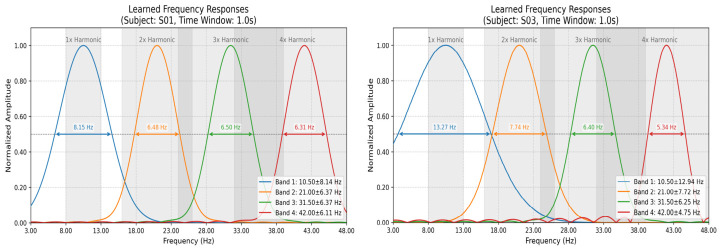
Frequency responses of Gabor filter banks learned for representative high- and low-performance subjects.

To visually demonstrate how G-AFB handles these inherent individual differences, we selected two representative subjects with the highest and lowest signal quality, S01 and S03, respectively. We then visualized the frequency-response curves of the G-AFB filter banks learned after training (Figure 10). This comparison illustrates the data-driven adaptive behavior of G-AFB.

For S01 (Figure 10, left), which had high signal quality, the SSVEP response showed a typical high-quality harmonic structure. Accordingly, G-AFB learned a regular and efficient filter bank. The center frequencies of the four subbands (10.50, 21.00, 31.50, and 42.00 Hz) closely matched the ideal harmonic relationship, and the bandwidths (full width at half maximum, FWHM) were relatively consistent and narrow (6–8 Hz). This indicates that the model could extract discriminative features from each harmonic with high confidence and precision.

By contrast, for S03 (Figure 10, right), which had poor signal quality, G-AFB learned an atypical filter bank characterized by adaptive compromise. The most evident difference was bandwidth adjustment: at the fundamental frequency, the model learned a much wider filter for S03 (FWHM ≈ 13.27 Hz) than for S01 (FWHM = 8.15 Hz). This is a typical adaptive strategy. When the fundamental-frequency response is unstable or has low SNR, the model widens the passband to capture signal energy over a broader range, thereby improving feature-extraction robustness.

### 3.3. Experiment 2: Validation of TriS-DANN (Cross-Subject Evaluation)

#### 3.3.1. Experimental Paradigm and Baseline Strategies

After validating G-AFB in the within-subject setting, we evaluated a more challenging and practically relevant scenario: cross-subject decoding. This experiment assessed the effectiveness and stability of TriS-DANN for achieving minimal calibration.

We adopted a leave-one-subject-out (LOSO) cross-subject evaluation strategy. For a dataset containing N subjects, each subject was selected in turn as the target domain, and the remaining N−1 subjects were combined as the source domain. To simulate a minimal-calibration process requiring limited user cooperation, the target-subject data were strictly partitioned: 21 trials were used as the only calibration data, another 21 independent trials were used as a validation set for model selection and early stopping without gradient updates, and the remaining 70 trials formed an independent test set for final performance evaluation.

Only the 21 target-domain calibration trials were used in the adaptation and fine-tuning stages. In Stage 2, these calibration trials were used without labels as unlabeled target-domain samples for domain alignment. In Stage 3, the same calibration trials were used with labels for supervised fine-tuning. The 21 validation trials were used only for model selection and early stopping, and the 70 test trials were used only for final performance evaluation.

To comprehensively evaluate the proposed method, we compared it with three benchmark strategies, all based on the best-performing G-AFB-tCNN architecture:

1. Baseline (pre-training only): This strategy quantifies cross-subject domain differences. A model pre-trained on the source domain is directly evaluated on the target-domain test set without any target-domain adaptation. Its performance is treated as the lower bound for transfer-learning methods.
2. Traditional fine-tuning (fine-tuning only): This strategy simulates conventional small-sample calibration. A source-domain pre-trained model is directly fine-tuned using 21 labeled calibration trials from the target domain.
3. Fully trained within-subject benchmark: This strategy provides the empirical upper bound for evaluating the gap between lightweight calibration and ideal subject-specific training. The benchmark corresponds to the G-AFB-tCNN results from Experiment 1, where each target subject was trained using all available training data (e.g., 91 trials in the in-house dataset).

The proposed TriS-DANN method follows the complete three-stage semi-supervised domain adaptation procedure: Stage 2 first performs unsupervised adaptation using the 21 calibration trials without labels, and Stage 3 then fine-tunes the model using the same 21 trials with labels. Model selection was based on the target-domain validation accuracy. During training, the checkpoint with the highest validation accuracy was saved as the final adapted model. The independent test set was not used for training, domain adaptation, fine-tuning, hyperparameter tuning, early stopping, or checkpoint selection. The hyperparameters for the three stages of cross-subject transfer learning are listed in Table 5.

#### 3.3.2. Performance Comparison and Analysis

**Figure 11:**
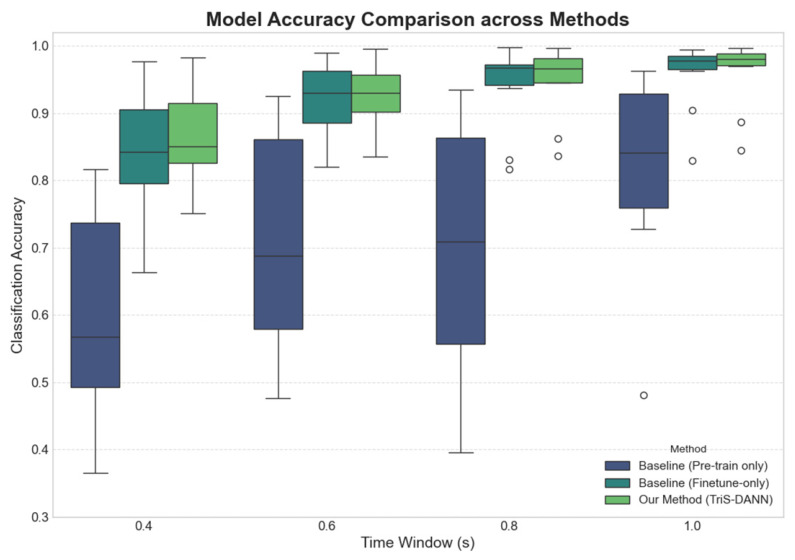
Box-and-whisker plot of the average accuracy for different cross-subject transfer strategies. The boxes represent the interquartile range, whiskers indicate the non-outlier range, and open circles denote outliers.

We systematically evaluated the average classification accuracy of different transfer strategies across multiple time windows. The results are shown in Table 6. To visualize performance stability, we further plotted the accuracy distribution of each strategy across all test subjects, as shown in Figure 11.

Combining Table 6 and Figure 11, several conclusions can be drawn. First, the source-only strategy performed worst, achieving only 60.27% accuracy in the most challenging 0.4 s window and showing very low stability, as indicated by the large variation in the box plot. This confirms that physiological differences among subjects are substantial and that direct model transfer is ineffective. Second, traditional fine-tuning substantially improved performance by using a small number of labeled samples, demonstrating the necessity of minimal calibration. However, its wider boxes and more outliers indicate larger inter-subject performance variability and limited stability.

By contrast, the proposed TriS-DANN strategy achieved the best balance between accuracy and stability. Its average classification accuracy exceeded that of traditional fine-tuning across all time windows. Moreover, the box plots show that TriS-DANN consistently produced shorter boxes and fewer outliers, indicating lower inter-subject variability. This stability is essential for plug-and-play BCI systems because it improves reliability for new users.

Finally, comparison with the fully trained within-subject benchmark indicates the gap between lightweight calibration and the empirical upper bound. Under the most challenging 0.4 s window, the accuracy of TriS-DANN (86.60%) was only 4.95 percentage points lower than the full-data benchmark (91.55%). Under the 1.0 s window, this gap decreased to 1.28 percentage points. Thus, the proposed strategy recovered most of the subject-specific performance while reducing the number of calibration trials by more than 76.9% (from 91 to 21), demonstrating its potential for practical low-calibration BCI systems.

#### 3.3.3. Verification of the Domain-Distribution Alignment Mechanism

The previous section demonstrated the performance advantage of TriS-DANN. To reveal the mechanism underlying this improvement, we further examined whether domain adaptation reduced the discrepancy between source- and target-domain feature distributions. Maximum mean discrepancy (MMD) was used for quantitative analysis, and t-distributed stochastic neighbor embedding (t-SNE) was used for qualitative visualization.

We first conducted a quantitative analysis. MMD is a non-parametric metric for measuring the discrepancy between two probability distributions. It maps samples into a high-dimensional reproducing-kernel Hilbert space (RKHS) through a kernel function and calculates the distance between the mean embeddings of the two distributions. A smaller MMD value indicates greater distributional similarity [32]. We calculated MMD values between the source- and target-domain feature distributions after Stage 1 pre-training (before adaptation) and after Stage 2 unsupervised domain adaptation (after adaptation). As shown in Table 7, MMD decreased under all time windows after unsupervised domain adaptation, with average reductions of 16.15%, 13.35%, 11.39%, and 15.50% at 0.4, 0.6, 0.8, and 1.0 s, respectively. These results provide quantitative evidence that adversarial unsupervised training effectively reduces the feature-distribution discrepancy between the source and target domains.

**Figure 12:**
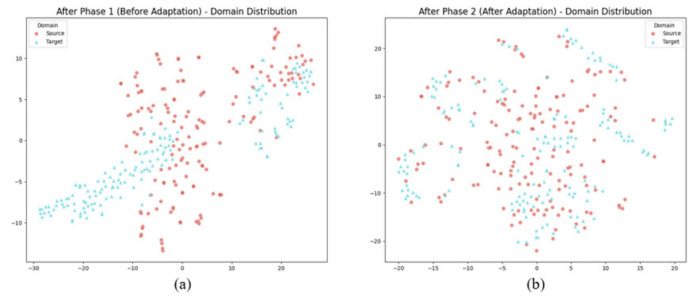
t-SNE feature distributions before and after unsupervised domain adaptation. (**a**) Before adaptation, source-domain and target-domain features are clearly separated. (**b**) After unsupervised domain adaptation, the two feature distributions become more aligned.

To visualize this process, t-SNE was used to project high-dimensional features extracted by the feature extractor into a two-dimensional space [33]. As shown in Figure 12a, before domain adaptation, source-domain features and target-domain features were clearly separated, forming distinct clusters and revealing a pronounced cross-subject domain gap. As shown in Figure 12b, after the second-stage domain-adversarial training, the two feature distributions overlapped substantially, indicating successful domain alignment.

In summary, both MMD-based quantitative analysis and t-SNE visualization confirm the effectiveness of TriS-DANN from the perspective of feature-space alignment. The key mechanism is the robust paradigm of unsupervised distribution pre-alignment followed by supervised fine-tuning. By reducing most domain differences in the feature space before fine-tuning, TriS-DANN provides a more domain-invariant starting point and enables efficient, stable personalized calibration using only a few labeled samples. This explains why TriS-DANN outperforms conventional fine-tuning in lightweight calibration scenarios.

## 4. Discussion

The preceding experiments systematically validated the effectiveness of G-AFB and TriS-DANN in improving SSVEP-BCI decoding performance and reducing calibration cost. This section discusses the mechanisms behind these findings, their implications, and the limitations and future directions of the study. We first analyze why G-AFB enables individualized adaptation and then discuss the role of the pre-alignment paradigm in lightweight calibration.

### 4.1. Mechanism of Individualized Adaptation in G-AFB

Experiment 1 showed that G-AFB can substantially improve within-subject decoding performance by enabling individualized adaptation. This advantage stems from its ability to respond to physiological differences among users. In our experiments, such differences were reflected by SNR variability; however, SNR is only one aspect of inter-subject differences. From a neurophysiological perspective, factors such as skull thickness, cortical structure, and neuronal organization may shift the energy distribution, phase characteristics, and optimal response frequency bands of SSVEP responses. Traditional fixed filter banks are based on a one-size-fits-all assumption and therefore cannot capture these subtle but important individual characteristics, limiting decoding performance. In contrast, the data-driven G-AFB layer can dynamically adjust its center frequency and bandwidth through end-to-end learning, thereby matching each user’s neural response pattern and improving decoding performance.

This interpretation is further supported by the visualization results for subjects with different signal quality. In high-SNR participants such as S01, the learned filters approached a regular harmonic structure, indicating that a fixed filter bank may already be effective under favorable recording conditions. By contrast, for the lower-SNR subject S03, G-AFB learned a wider passband at the fundamental frequency, suggesting adaptive compensation for weaker or less stable SSVEP responses. Thus, G-AFB may be particularly useful when EEG quality is unstable or individual variability is pronounced. This property may be relevant to populations with greater neurophysiological heterogeneity, such as patients with neurodegenerative diseases or stroke, although further clinical validation is required.

### 4.2. Role of the Pre-Alignment Paradigm in Lightweight Calibration

Experiment 2 showed that TriS-DANN outperformed conventional fine-tuning in both accuracy and stability under the lightweight calibration setting. This advantage arises from its pre-alignment paradigm: unsupervised domain alignment followed by supervised fine-tuning. Conventional fine-tuning is simple but assumes that a small number of labeled target-domain samples can compensate for source-target feature-distribution differences. In practice, this assumption is risky. When the domain shift is large, the model may overfit the limited target samples and learn features with poor generalizability. In addition, optimization is performed in a feature space distorted by domain shift, which can lead to local optima and large performance fluctuations across subjects.

By contrast, the unsupervised domain-adaptation stage in TriS-DANN directly addresses feature-space distortion. Through domain-adversarial training, the model learns domain-invariant representations and reduces source-target differences at the feature level. As illustrated by the t-SNE results in Figure 12, features that were initially separated become aligned after adaptation. This pre-alignment step provides a smoother and more general feature space for subsequent fine-tuning. In this aligned space, a small number of labeled target-user samples can more effectively guide the model to learn personalized features on top of common representations, instead of using most of their supervisory information to overcome domain mismatch. This mechanism explains why TriS-DANN uses limited calibration data more efficiently and achieves more stable lightweight calibration.

### 4.3. Limitations and Future Work

It should be noted that the in-house short-window results should not be interpreted as direct evidence of onset-locked rapid online BCI operation. The in-house experiment used a block-based long-stimulation design, and the 0.4 s and 1.0 s samples were extracted from the 12.0 s analysis epoch after visual-latency correction. Therefore, these samples may correspond to stabilized SSVEP responses rather than the earliest post-stimulus response. The present results demonstrate the ability of the proposed method to classify short segments extracted from long-duration SSVEP responses, but further validation using a strict trial-by-trial online paradigm and onset-locked windows is required to evaluate true rapid SSVEP-BCI decoding.

## 5. Conclusions

This study addressed two major challenges in SSVEP-BCI systems: substantial inter-subject variability and weak cross-subject generalization, which together lead to high calibration costs. We proposed and validated an end-to-end decoding framework that integrates a Gabor adaptive filter bank (G-AFB) with a three-stage semi-supervised domain adaptation network (TriS-DANN).

First, for feature extraction, we designed an end-to-end trainable G-AFB module. Within-subject experiments demonstrated that G-AFB can learn individualized filter banks that match the neural response characteristics of different users. Compared with conventional fixed filtering, the proposed adaptive filtering method significantly improved performance on both datasets.

Second, for model generalization, we designed and evaluated the TriS-DANN lightweight calibration strategy. Cross-subject experiments showed that, by combining unsupervised distribution pre-alignment with supervised fine-tuning, TriS-DANN outperformed conventional fine-tuning in both classification accuracy and stability using only 21 calibration trials. Its decoding performance approached the empirical upper bound obtained by within-subject training with full data.

Finally, by including an idle state in the task design, we verified the robustness of the proposed framework under conditions closer to practical applications. The results indicate that the framework can reliably distinguish intentional commands from non-control states.

Overall, this study demonstrates that jointly optimizing adaptive frequency-band learning and semi-supervised feature alignment is an effective strategy for constructing practical SSVEP-BCI systems with high performance, high reliability, and low calibration cost. The proposed framework provides a technical basis and feasible implementation route for translating SSVEP-BCI technology from laboratory research to broader practical use.

## Figures and Tables

**Table 1:** Architecture of the G-AFB-tCNN network.

Layer Type	Output Shape	Description
Input	C × T × 1	C: Channels, T: sample points
G-AFB Layer	C × T × S × 1	S: number of subbands = 4
Parallel Sub-band Processing		
Slicing (per subband)	C × T × 1	Slice a single sub-band from G-AFB output
Conv2D	(C-7) × T × 16	Filters = 16, Kernel = (8, 1), Padding = ‘valid’
BatchNormalization	(C-7) × T × 16	
Activation	(C-7) × T × 16	elu
Dropout	(C-7) × T × 16	Rate = 0.4
Conv2D	(C-7) × [T/5] × 16	Filters = 16, Kernel = (1, 5), Strides = (1, 5)
BatchNormalization	(C-7) × [T/5] × 16	
Activation	(C-7) × [T/5] × 16	elu
Dropout	(C-7) × T × 16	Rate = 0.4
Conv2D	(C-7) × ([T/5]-4) × 16	Filters = 16, Kernel = (1, 5), Strides = (1, 5)
BatchNormalization	(C-7) × ([T/5]-4) × 16	
Activation	(C-7) × ([T/5]-4) × 16	elu
Conv2D	(C-7) × ([T/5]-4) × 64	Filters = 64, Kernel = (1, 5), Strides = (1, 5)
BatchNormalization	(C-7) × ([T/5]-4) × 64	
Activation	(C-7) × ([T/5]-4) × 64	elu
Flatten	F	F = (C-7) × ([T/5]-4) × 64
Concatenate	S × F	Concatenate features from all S sub-bands
Classification Head		
Dense	256	Units = 256; activation = ‘elu’
Dropout	256	Rate = 0.4
Dense (Output)	k	Units = k(number of classes),Activation = ‘softmax’

**Table 2:** Experimental hardware platform.

Item	Value
Operating System	Windows 11
CPU	Intel(R) Core(TM) i5-12400F
GPU	NVIDIA GeForce RTX 4070
RAM	12 GB
Programming language	Python 3.9
Machine Learning Platform	Tensorflow 2.7

**Table 3:** Hyperparameters for within-subject evaluation.

Hyperparameter	Value
Learning rate	0.001
Optimizer	Adam (momentum = 0.99)
Batch Size	500
Dropout Rate	0.25
Training epochs	600
L2 regularization	0.0001

**Table 4:** Model evaluation results on the public dataset.

Base Model	Filtering Strategy	Mean Accuracy (%)	Standard Deviation (%)	Improvement Over Original
	Original	77.38	6.23	—
tCNN	FB	84.50	4.96	+7.12 pp
	**G-AFB**	**89.13**	**4.13**	**+11.75 pp**
	Original	80.13	6.03	—
EEGNet	FB	86.99	4.61	+6.86 pp
	**G-AFB**	**82.53**	**5.60**	**+2.40 pp**
	Original	80.54	4.16	—
SSVEPFormer	FB	84.05	4.48	+3.51 pp
	**G-AFB**	**84.71**	**4.74**	**+4.17 pp**

Note: Bold values indicate the results obtained using the proposed G-AFB filtering strategy.

**Table 5:** Three-stage hyperparameters for TriS-DANN cross-subject transfer learning.

Hyperparameter	Stage 1 (Source-Domain Pre-Training)	Stage 2 (Unsupervised Adaptation)	Stage 3 (Few-Label Fine-Tuning)
Feature-extractor learning rate	1 × 10^−2^	1 × 10^−4^	1 × 10^−5^
Label-classifier learning rate	1 × 10^−2^	N/A (frozen)	1 × 10^−5^
Domain-classifier learning rate	N/A	1 × 10^−4^	N/A
Domain loss weight (λ_d_)	N/A	0.5	N/A
Batch size	256	256 (source domain) + 256 (target domain)	64 (target domain only)
Training epochs	250	60	40

**Table 6:** Average classification accuracy of different transfer strategies across time windows.

Signal Length	0.4 s	0.6 s	0.8 s	1.0 s
Source only [25]	60.27 ± 14.95%	70.84 ± 16.15%	70.36 ± 18.88%	81.97 ± 14.44%
Fine-tuning only [31]	84.57 ± 9.21%	91.78 ± 6.05%	93.49 ± 6.28%	95.76 ± 5.15%
TriS-DANN	86.60 ± 7.16%	92.28 ± 5.40%	94.67 ± 5.43%	95.98 ± 5.11%
Full-data within-subject benchmark	91.55 ± 5.44%	94.90 ± 6.71%	96.78 ± 5.30%	97.26 ± 5.19%

**Table 7:** Average MMD values and reduction rates before and after domain adaptation under different time windows.

Signal Length	MMD Before Adaptation	MMD After Adaptation	MMD Reduction (%)
0.4 s	0.0246	0.0206	16.15%
0.6 s	0.0191	0.0166	13.35%
0.8 s	0.0167	0.0148	11.39%
1.0 s	0.0162	0.0136	15.50%

## Data Availability

The public SSVEP benchmark dataset used in this study is available from the Tsinghua BCI Lab download page (https://bci.med.tsinghua.edu.cn/download.html (accessed on 10 March 2026)). The source code of the proposed framework, including the implementation of G-AFB-tCNN and TriS-DANN, related training scripts, evaluation scripts, and configuration files, may be made available from the corresponding author upon reasonable request for academic and reproducibility purposes. The in-house EEG dataset is not publicly released because it was collected as part of an ongoing research project and contains human-subject EEG recordings. Access to the in-house data may be provided upon reasonable request, subject to institutional approval, project-related restrictions, and participant privacy protection requirements.

## Abbreviations

The following abbreviations are used in this manuscript:

BCI	Brain–computer interface
SSVEP	Steady-state visual evoked potential
EEG	Electroencephalography
G-AFB	Gabor adaptive filter bank
TriS-DANN	Three-stage semi-supervised domain-adversarial neural network
CNN	Convolutional neural network
CCA	Canonical correlation analysis
ITR	Information transfer rate
